# Analysis of Protein Disorder Predictions in the Light of a Protein Structural Alphabet

**DOI:** 10.3390/biom10071080

**Published:** 2020-07-20

**Authors:** Alexandre G. de Brevern

**Affiliations:** INSERM, UMR_S 1134, DSIMB, Univ Paris, INTS, Laboratoire d’Excellence GR-Ex, 75015 Paris, France; alexandre.debrevern@univ-paris-diderot.fr; Tel.: +33-1-44493000

**Keywords:** protein structures, intrinsic disorder proteins, intrinsic disorder regions, X-ray structures, nuclear magnetic resonance, molecular dynamics, protein blocks, protein flexibility, mobility

## Abstract

Intrinsically-disordered protein (IDP) characterization was an amazing change of paradigm in our classical sequence-structure-function theory. Moreover, IDPs are over-represented in major disease pathways and are now often targeted using small molecules for therapeutic purposes. This has had created a complex continuum from order-that encompasses rigid and flexible regions-to disorder regions; the latter being not accessible through classical crystallographic methodologies. In X-ray structures, the notion of order is dictated by access to resolved atom positions, providing rigidity and flexibility information with low and high experimental B-factors, while disorder is associated with the missing (non-resolved) residues. Nonetheless, some rigid regions can be found in disorder regions. Using ensembles of IDPs, their local conformations were analyzed in the light of a structural alphabet. An entropy index derived from this structural alphabet allowed us to propose a continuum of states from rigidity to flexibility and finally disorder. In this study, the analysis was extended to comparing these results to disorder predictions, underlying a limited correlation, and so opening new ideas to characterize and predict disorder.

## 1. Introduction

Analyses of protein structures have been extensively done in light of classical repetitive secondary structures, namely the α-helices and the β-sheets, connected by loops [1]. Since the end of the 1980s, more complex structural alphabets (SAs) entirely describing protein structures, have been developed [2,3] and applied to multiple tasks e.g., protein superimposition [4,5,6], molecular dynamics analysis [7], binding site detections [8], flexibility prediction [9], or threading approaches [10,11]. In this field, protein blocks (PBs), structural alphabets have been particularly successful [12]. Recently, they have been used to analyze flexibility of protein structures [13,14] and molecular dynamics simulations [15], e.g., integrins [16], HIV-1 capsid protein [17], and the N-methyl-D-aspartate receptor (NMDA) receptor channel gate [18]. Nonetheless, all these studies did not look at an essential part of the proteins. They only focused on ordered regions.

Intrinsic disorder is characterized by lack of stable tertiary structure under physiological conditions [19]. Approximately 19% of residues in these proteins are disordered and essential for multiple cellular functions that involve protein-protein, protein-nucleic acid, and virus-host interactions [20]. The intrinsically-disordered proteins (IDPs) [21,22,23,24] do not have a well-defined 3-D structure but rather adopt an ensemble of conformations that are functional in solution [25]. IDPs exist as dynamic ensembles, within which atom positions and backbone angles exhibit random temporal fluctuations [26,27].

Both experimental and computational approaches have been used to address the questions of IDPs. Nuclear magnetic resonance (NMR) provides quantitative residue-level information on structure and dynamics of IDPs as structural ensembles [28]. Small-angle X-ray scattering (SAXS) and electron microscopy (EM) gives an in-situ ensemble model describing the conformational behavior of the disordered region. Molecular dynamics (MDs) is used to refine and propose such ensembles [29,30,31].

Another important point is that the classical sequence-structure-function dogma was questioned by these extremely fast movements and the rigid, flexible, and deformable dynamic states that it may acquire or lose [32,33], but is can be also be a patchwork of ordered and disordered regions [34].

Hence, within the analyses of flexibility in globular proteins, PBs were applied to an ensemble of structural models of IDPs, provided in a dedicated database named PED^3^ (Protein Ensemble Database) [35]. Using *N_eq_*, an entropy index derived from this structural alphabet, we underlined its interest to measure these local dynamics, and to quantify the continuum of states from rigidity to flexibility, and finally disorder. Non-disordered regions in the ensemble of disordered proteins were also underlined [36,37]. PBs had been efficiently used to highlight rigid local domains within flexible regions and so discriminate deformability and mobility concepts. To access IDP structural information is sometimes complex. A large number of prediction approaches exist. A remaining question was the question of the relationship between this new quantification and the quality of disorder prediction approaches. Indeed, through our different researches, rigid regions (i.e., mobile regions) were underlined between two flexible regions (i.e., deformable regions); these domains can be interpreted as disordered when they are not entirely disordered. This peculiarity can bias the prediction of disorder regions. In this study, two distinct prediction methodologies were used in this way and the correlation named *N_eq_* was evaluated.

## 2. Materials and Methods

### 2.1. Dataset

The IDP ensembles of structures were taken from Protein Ensemble Database (PED^3^). This database of conformational ensembles describes flexible proteins (http://pedb.vib.be/index.php, accessed on 25 May 2020) [35] and has 24 entries. The different IDPs came from different techniques, i.e., SAXS and NMR, NMR alone, SAXS alone and molecular dynamics [38,39,40,41,42,43,44,45,46,47,48] and have been analyzed in [36].

### 2.2. N_eq_ Entropy Index

Protein blocks (PBs) are the most widely-used structural alphabet composed of 16 local prototypes [12]. It is employed to analyze local conformations of protein structures from the Protein Data Bank (PDB) [49] (see [3] for a review of the structural alphabet and [50] for information on PBs). Each PB is characterized by the φ and ψ dihedral angles of five consecutive residues. PBs give a reasonable approximation of all local protein 3D structures and are very efficient for analyzing protein flexibility, i.e., molecular dynamics (MDs) analyses [16,18,51]. PB assignment was carried out for every residue from every structure/structural model extracted from PED^3^ using the PBxplore tool [52]. To quantify local protein flexibility, PB-derived entropy measure *N_eq_* (equivalent number of PBs) [12] was used. It represents the average number of PBs a residue may adopt at a given position. *N_eq_* is calculated as follows [12]:

(1) $$N_{eq}=exp(-\sum_{x=1}^{16}f_{x}lnf_{x})$$

where, *f_x_* is the frequency of PB *x* at the position of interest. *N_eq_* value can vary between 1 and 16. A *N_eq_* value of 1 indicates that only one type of PB is observed, while a value of 16 indicates an equal probability for each of the 16 PBs, i.e., random distribution.

### 2.3. Disorder Prediction from the Sequence

Two different approaches were initially used to predict protein disorder from the sole information of the sequence, namely Disopred3 (used through http://bioinf.cs.ucl.ac.uk/psipred/webserver) [53,54] and Protein DisOrder Prediction System (PrDOS, through http://prdos.hgc.jp/cgi-bin/top.cgi) [55], see [37]. Disopred3 combined two predictors of intrinsic disorder, one module that combined the intermediate results and one component that annotated protein-binding IDRs; their neural networks used evolutionary information encoded in a position-specific score matrix (PSSM) [53]. PrDOS also combined two separate predictors; the first one was a support vector machine algorithm using evolutionary information again with PSSMs. The second one assumed the conservation of intrinsic disorder in protein families [55]. Results were finally also compared to a physics-based approach. IUPred2A and ANCHOR2 were used [56]. IUPred2A used an energy estimation method at its core. This approach utilized a low-resolution statistical potential to characterize the tendencies of amino acid pairs to form contacts, observed in a collection of globular protein structures. ANCHOR2 followed the same principle for disordered binding regions [57].

A prediction rate was used in the analysis. This prediction rate used (a) the predicted state (order and disorder) taken directly from Disopred3 and PrDOS and (b) the ‘true’ state defined by *N_eq_*, i.e., if the *N_eq_* is lower that the threshold value, it is an order state while if it is higher, it is a disorder state.

### 2.4. Analyses

The analyses were done using Python programming language v.2.7.10 [58], and R software v.3.3.3 [59] while 3D visualization was done using MacPyMOL software v.1.7.2.2 [60,61]. Different correlations are calculated, they are all based on the use of values coming from Disopred3 and/or PrDOS (in a range from 0 to 1), or with *N_eq_* (in a range from 1 to 16), and all done with continuous values and not binary classes. A perfect correlation is equal to 1.0, while a value of 0 corresponds to an absence of correlation.

## 3. Results

### 3.1. Data Analyses

**Figure 1:**
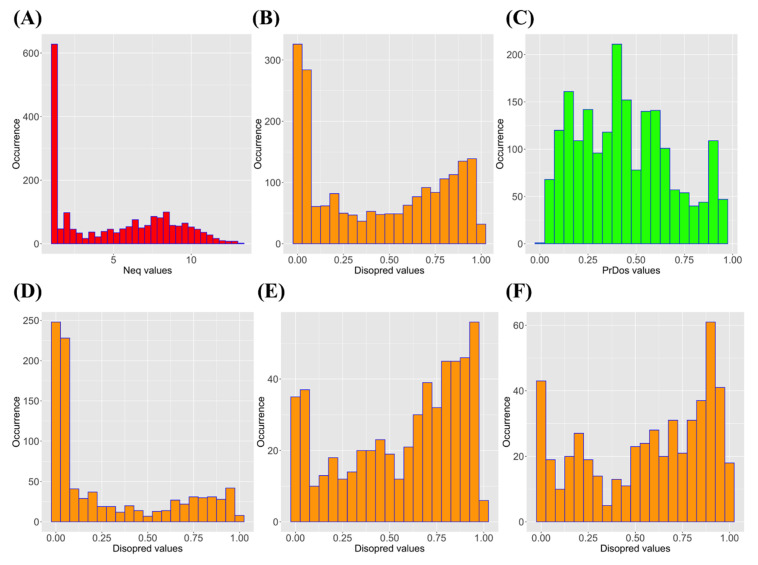
Distribution of an equivalent number of PBs (*N*_eq)_ and prediction disorder values. (**A**) *N_eq_* values (in red), (**B**) Disopred3 values (in orange), (**C**) PrDOS values (in green), (**D**) Disopred3 values for *N_eq_* values lower than 4, (**E**) Disopred3 values for *N_eq_* values between 4 and 8, and (**F**) Disopred3 values for *N_eq_* values higher than 8.

The PED^3^ database encompasses an interesting set of structural behaviors. Figure 1A shows the *N_eq_* distribution. 58% of the positions are entirely rigid with a *N_eq_* of 1.0. In previous studies, we have analyzed ordered structures using a large dataset of molecular dynamics simulations. Hence, this number is equivalent to the one observed for ordered structures (60% for *N_eq_* of 1.0) [62]. However, these behaviors between order and disorder dataset evolved very rapidly. The disorder state had 36% of residues with an *N_eq_* higher 2 when it was only 8% for ordered structures. An *N_eq_* higher than 8 can be considered as entirely disordered state. The PED^3^ database had 15% of its residues with this feature while there were only 0.01% in ordered structures. The PED^3^ database allowed a very interesting continuum from rigidity to flexibility to high flexibility and finally disorder [36].

In protein structures obtained from X-ray crystallography, the definition of a disordered region can be considered as quite simple as it is linked to the absence of the atoms in a specific part of the sequence, i.e., they cannot be trapped as they are moving too fast [22]. Several physico-chemical and structural properties of intrinsic disorder are now well established (e.g., high net-charge and low hydrophobicity), and a large number of prediction methodologies use these physico-chemical properties linked with machine learning and evolutionary information [63,64].

Two distinct approaches were used to perform protein disorder predictions from the sole information of the protein sequences, (i) Disopred3 (Figure 1B) [53] and (ii) PrDOS (Figure 1C) [55].

**Figure 2:**
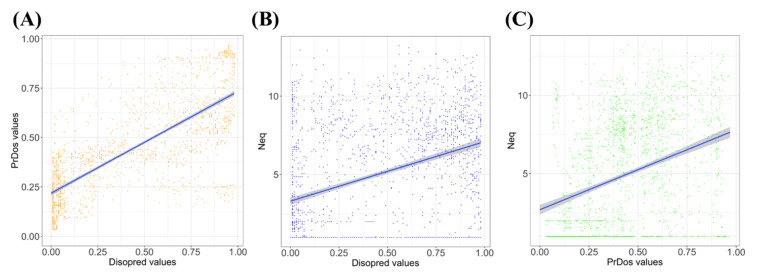
*N_eq_* versus prediction disorder results. (**A**) Disopred3 values (*x*-axis) against PrDOS values (*y*-axis) (correlation equals to 0.75), (**B**) *N_eq_* values (*x*-axis) against Disopred3 values (*y*-axis) (correlation equals to 0.37) and (**C**) *N_eq_* values (*x*-axis) against PrDOS values (*y*-axis) (correlation equals to 0.34).

The two distributions were quite different with more extreme values for Disopred3 and had a more centered distribution for PrDOS (Figure 1B,C). From their raw values, both methods also proposed the order/disorder two-states prediction, with 81.1% of predictions in common (32.9% of common disordered predicted positions and 48.2% of ordered ones). Disopred3 predicted more disordered positions (46.1%) than PrDOS (38.6%). They had a correct correlation of 0.75 in regard to the differences in the distribution of predicted values (Figure 2A).

### 3.2. Comparison of N_eq_ with Prediction Results

As previously shown, analysis with local protein conformation is often different from global analysis. For instance, analyses of molecular dynamics simulation showed large difference between RMSf and *N_eq_* [13,16,18,65], with the correlation being slightly higher than 0.4. Therefore, the correlation between *N_eq_* and the prediction values was not expected to be much better. Moreover, the distributions of both structural information (namely *N*_eq_, see Figure 1A) and disorder predicted values (Figure 1B,C) were not well spread. Hence, the correlation of *N_eq_* and Disopred3 was 0.37 (Figure 2B), and of *N_eq_* and PrDOS it was 0.34 (Figure 2C).

It is possible to divide *N_eq_* values into different clusters to have a better view of the prediction methods. *N_eq_* values of less than 4 correspond to the most rigid and the least flexible positions. Corresponding prediction values of Disopred3 and PrDOS were, respectively, 0.28 (Figure 1D) and 0.35 (Figure S1A). For intermediate *N_eq_* values (between 4 and 8, corresponding to flexible regions to the border of disorder [36]), average prediction values were 0.57 (Figure 1E) and 0.48 (Figure S1B), respectively. While for the disorder region (*N_eq_* higher than 8), these prediction values were 0.56 (Figure 1E) and 0.54 (Figure S1C), respectively.

These results showed clear and significant differences. Interestingly, both prediction methods displayed different distribution values, but often went to the same predictions and behaviors. For instance, in the disorder region (*N_eq_* higher than 8), their correlation was still excellent (0.76), as they both predicted high (disorder) and low (order) values (Figure S2).

### 3.3. General Tendencies

**Figure 3:**
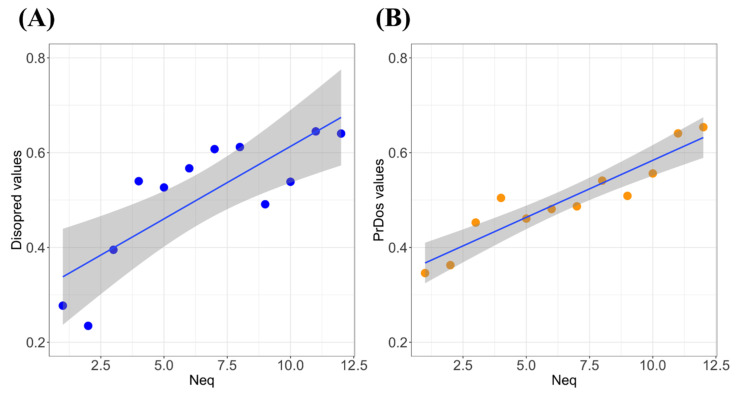
Analyses of *N_eq_* versus prediction disorder results on PED^3^ dataset. (**A**) *N_eq_* values (*x*-axis) vs. average Disopred3 values (*y*-axis) (correlation is of 0.81) and (**B**) *N_eq_* values (*X*-axis) vs. average PrDOS values (*y*-axis) (correlation is of 0.93).

Previous analyses underlined the distribution of predicted values of Disopred3 and PrDOS, in regard to this new quantification. Figure 3 shows a similar computation, but these were done with the average values per class of *N_eq_*. Classes were designed for twelve values ranging from 1.0 to 12.0 (and higher). As often seen, the correlations became largely better using average values, i.e., the correlation is of 0.81 for Disopred3 (Figure 3A) and of 0.93 for PrDOS (Figure 3B).

These correlations looked good but did not reflect entirely the sensitivity of each method. Indeed, the excellent correlation of PrDOS came mainly from the limited variation of the predicted values (Figure 1C and Figure S3B) while Disopred3 was more pertinent.

## 4. Discussion and Conclusions

Intrinsically-disordered proteins and regions are complicated, as they do not have unique and simple characteristics. Hence, IDPs represent complete disordered proteins that stay disordered, but they can also adopt one conformation when they bind to their ligands or partners [66,67] or participate in multiple systems [68], they are essential to functions [69,70], drug design [71], and protein design [72].

For instance, NMR spectroscopy was used to delineate the sites of pre-structured motifs (PreSMos) [73]. PreSMos are transient local structural elements that presage target-bound conformations and act as specific determinants for IDP recognition by their target proteins [74]. Related to PreSMos are the famous molecular recognition features (MoRFs) that were identified in the x-ray structures of complexes [75] between target proteins and short fragments of IDPs/IDRs (predicted to be disordered) [76,77,78]. They were also linked to short linear motifs (SLiMs) that were found four times out of five in IDRs. The lengths of SLiMs range from 3 to 11 residues, and they have often been associated with pathologies, and characterized as structured when they interact with repetitive structures [79,80].

In previous studies, it was observed that local protein conformation deformation could be quantified and defined more properly in terms of rigidity and flexibility [15,62]. The analyses were extended to IDPs leading to a definition of an entropy scale ranging between 1 and 16, i.e., the number of PBs. An *N_eq_* of 1 corresponded to a rigid position while 8 was a disordered one, extending this categorization led to a *N_eq_* of 4 for a flexible region and of 6 for a highly flexible region. The analysis of IDPs also underlined a large number of IDRs and rigid regions [36]. *N_eq_* was a great tool to locate the mobile region encompassed in deformable (flexible) regions, and this was the same mechanism in IDPs. The question raised was then the reaction of disorder predictions on these data.

Intrinsic disorder predictors have been created from a wide variety of architectures and data sets with three main categories based on their underlying models: (a) ab initio methods based on the physiochemical characteristics, (b) machine-learning methods, such as PrDOS [55], and (c) meta methods, such as Disopred3 [53]. The relative performance of intrinsic disorder predictors has been compared many times [64,81] and we note the recent DISOselect analyses [82]. These all underlined the specificity of each approach, with two different methodologies were used here.

Firstly, Disopred3 is a complex approach to performing protein disorder prediction and protein-binding site annotation within disordered regions [53]. The tool first identifies disordered residues through a consensus of the output generated by Disopred2 [83] and two additional machine-learning-based modules trained on large IDRs. It then annotates them as protein binding through an additional support vector machine (SVM) classifier. Secondly, PrDOS [55] is composed of two predictors: (i) a predictor based on the local amino acid sequence, using a SVM algorithm for the position-specific score matrix, and (ii) one based on template proteins with the use of the conservation of intrinsic disorder in protein families using the Position specific iterative-Basic Local Alignment Search Tool (PSI-BLAST) approach [84].

As seen with Figure 1 and Figure 2, both approaches presented similar tendencies with a strong difference in the range of predicted values. The final correlations with *N_eq_* were limited. These two prediction methodologies were mainly controlled by the use of evolutionary information with effective neural networks. To go further, physics-based approaches were used, namely IUPred2A and ANCHOR2 [56]. Even if IUPred2A showed a distribution of prediction values close to the distribution of Disopred3 (Figure S6A), ANCHOR2 displayed a surprising binomial distribution with few values close to zero (Figure S6B). The correlations of IUPred2A and ANCHOR2 with *N_eq_* were particularly weak (0.29 and 0.25), leading to no particular improvements (Figure S6C,D). These approaches showed specificities, as (i) they had a correlation of 0.79 between them, but (ii) weak correlations with Disopred3 and PrDOS (between 0.65 and 0.44, see Table S1), underlying specific prediction patterns.

**Figure 4:**
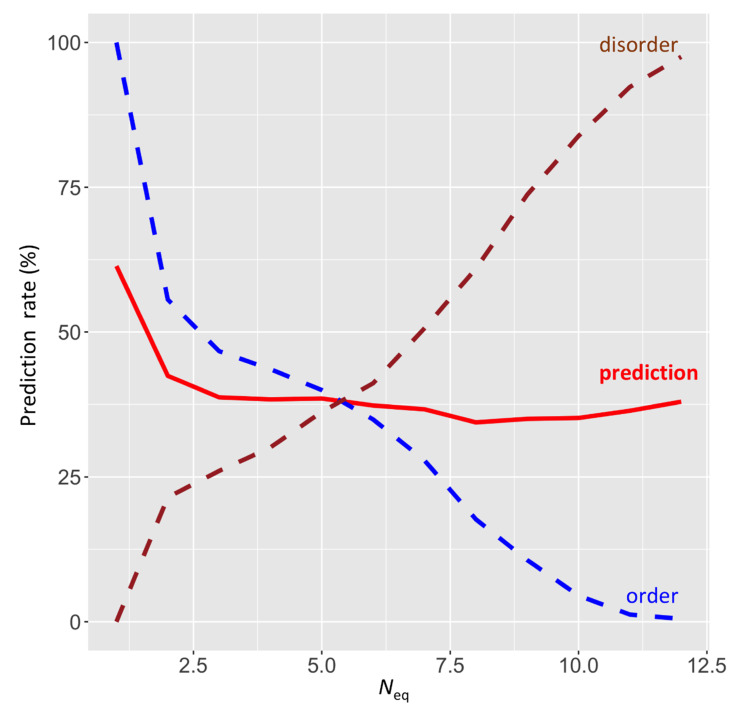
Evaluation of Disopred3 predictions at the light of *N_eq_* values. *N_eq_* values ranging from 1.0 to 12 are provided with the prediction rate (in red). The contribution of disordered and ordered positions are defined by the *N_eq_* values.

To go further, *N_eq_* was used to evaluate the quality of disorder prediction; the limit between order and disorder was defined by *N_eq_* (ranging between 1.0 and 12). Figure 4 summarizes the results with a limited success mainly between 35% and 42% (Table S2). These results do not mean that the prediction methods are of poor quality, but that a dedicated (and well-equilibrated) dataset must be used ([85]), with also well-balanced order and disorder state distribution. For instance, better prediction for *N_eq_* of 1 is due to a great imbalance for predicted order state. It is also a direct reflection of the fact that *N_eq_* is a particular measure and that we need the development of a specific prediction approach, such as OPAL+ which is specialized for MoRF prediction, to predict this type of feature [75].

**Figure 5:**
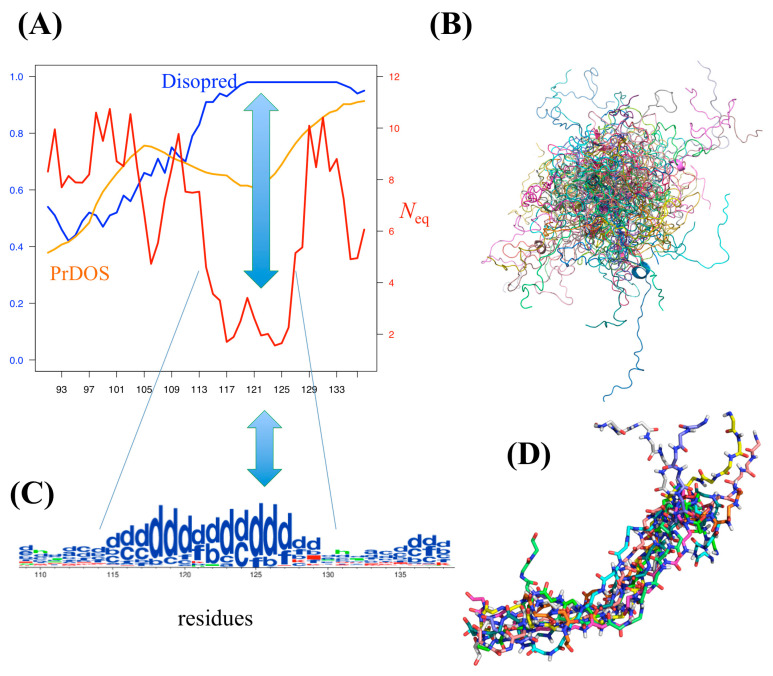
Example of alpha-synuclein, a solution-state ensemble from paramagnetic relaxation enhancement-Nuclear magnetic resonance (PRE-NMR) ensemble-restrained MD simulations (PED^3^ 9AAC entry). (**A**) A zoom on C-terminus of alpha-synuclein was done. Along the *x*-axis are shown, in orange, PrDOS prediction values and, in blue, Disopred3values. *N_eq_* values are represented in red. (**B**) A superimposition of hundreds of structural models from the ensemble. (**C**) Representation in terms of PBs shown with WebLogo [86], underlying the most contradictory position (low *N_eq_* and high disorder prediction values). (**D**) A dozen structural models superimposed on this region.

As an interesting example, PED^3^ 9AAC entry corresponded to alpha-synuclein. In its C-terminal part, both PrDOS and Disopred3 predicted disordered and highly disordered regions (orange and blue colors, respectively, on Figure 5A). The tendencies are similar with IUPred2A, ANCHOR2 [56], and the approaches proposed in DECIPHER [70]), while *N_eq_* was around 2 at these positions (red color on Figure 5A), i.e., a quite rigid region. Indeed, the visualization of the protein ensemble (Figure 5B) does not show any ordered regions, while PB distribution showed (Figure 5C, Figures S4 and S5) a large proportion of PB *d* around this region, i.e., a curved region (Figure 5D).

This type of example highlighted the interest in going further on a large dataset of IDPs and IDRs to present a better view of these cases. In the future, we would like to integrate different types of information to propose such methodologies that could be useful in different research areas. The PED^3^ database is an excellent source for analyses of both order and disorder states, with entirely disordered proteins and proteins with IDRs, i.e., mimicking a large spectrum of protein behaviors. Nonetheless, the number of data is still limited and it would be of great interest to add more information coming from other sources, computational and/or experimental.

In summary, analyses of IDPs in the light of structural alphabet underlined specific behaviors that were slightly different from classical IDPs/IDRs and the need to go further to find out if specific approaches and methodologies could be applied to it. It is therefore possible to define specific prediction methodologies that will separate mobile regions from pure disorder regions, i.e., rigid zones encompassed in highly-flexible deformable regions. It seems manageable, as we have seen that correlations with classical disorder predictions are not great (around 0.3, see Figure 2), but when average values are taken into account (Figure 3), correlations are clearly better (around 0.9).

## Supplementary Materials

The following are available online at https://www.mdpi.com/2218-273X/10/7/1080/s1, Figure S1: Prediction disorder results of PrDOS on PED^3^ dataset. For *N_eq_* (A) lower than 4. (B) between 4 and 8 and (C) higher than 8. Figure S2: Prediction disorder results on PED^3^ dataset. (A) DisoPred3values (*x*-axis) against PrDOS values (*y*-axis) for *N_eq_* values higher than 8 (correlation is of 0.76). Figure S3: Analyses of prediction disorder results on PED^3^ dataset per class of *N_eq_*. (A) DisoPred3values (*x*-axis) and *N_eq_* classes (*y*-axis). (B) PrDOS values (*x*-axis) and *N_eq_* classes (*y*-axis). Figure S4: *N_eq_* values for the alpha-synuclein, a solution-state ensemble from PRE-NMR ensemble-restrained MD simulations (PED^3^ 9AAC entry). Computation done with PBxplore software. Figure S5: PB distribution for the alpha-synuclein, a solution-state ensemble from PRE-NMR ensemble- restrained MD simulations (PED^3^ 9AAC entry). Computation done with PBxplore software and represented with WebLogo. Figure S6: Distribution of *N_eq_* and prediction disorder values & *N_eq_* versus prediction disorder results. Distribution of (A) IUPred2A values (in purple), and (B) ANCHOR2 values (in pink), (C) *N_eq_* values (*x*-axis) against IUPred2A values (*y*-axis) (correlation equals to 0.29). (C) *N_eq_* values (*x*-axis) against ANCHOR2 values (*y*-axis) (correlation equals to 0.25). Table S1: Correlation between *N*_eq_, Disopred3, PrDOS, IUPRed2A and ANCHOR2 values on PED3 dataset. Table S2: Evaluation of prediction rate according to *N_eq_* values for Disopred3 approach.
