# Supplementary material for: Analysis of Protein Disorder Predictions in the Light of a Protein Structural Alphabet
Source: Biomolecules. 2020 Jul 20;10(7):1080. doi: 10.3390/biom10071080 (PMC7408373; doi:10.3390/biom10071080)
Supplement: Supplementary file 1 [file biomolecules-10-01080-s001.pdf]

Supplementary Material

Title: Analysis of Protein Disorder Predictions in the Light of a Protein Structural Alphabet

Author: Alexandre G. de Brevern

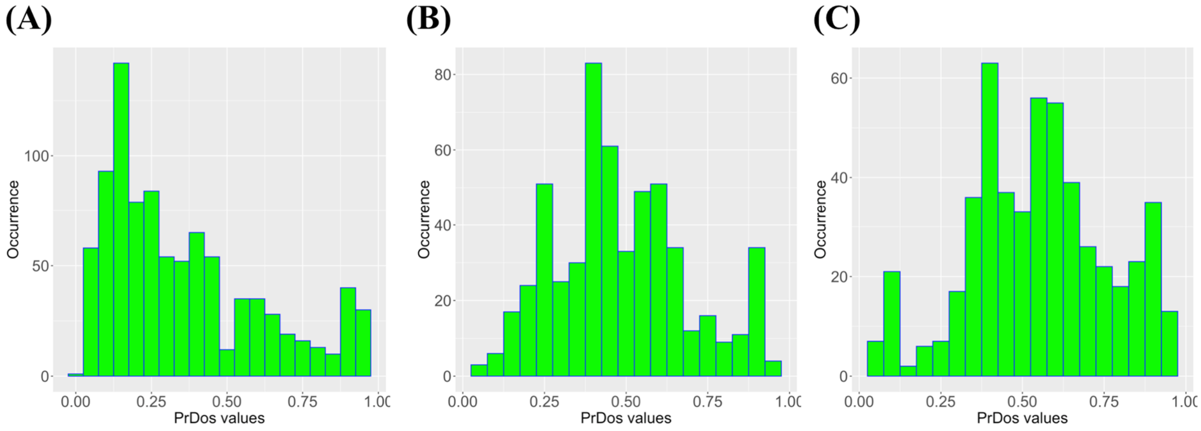

Figure S1. Prediction disorder results of PrDOS on PED[3] dataset. For N_{eq} (A) lower than 4, (B) between 4 and 8 and (C) higher than 8.

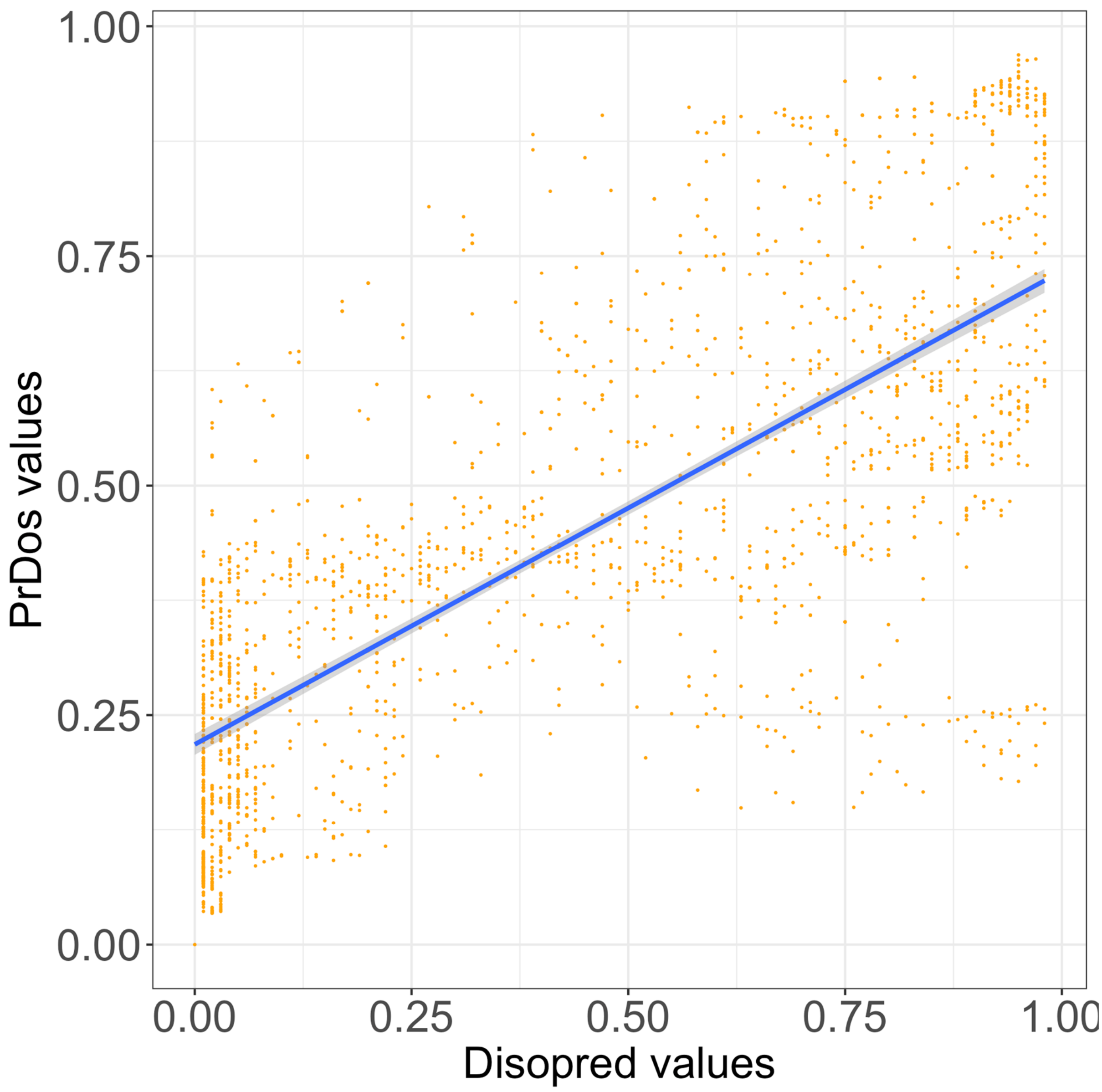

Figure S2. Prediction disorder results on PED^3 dataset. (A) DisoPred3 values (x-axis) against PrDOS values (y-axis) for N_{eq} values higher than 8 (correlation is of 0.76).

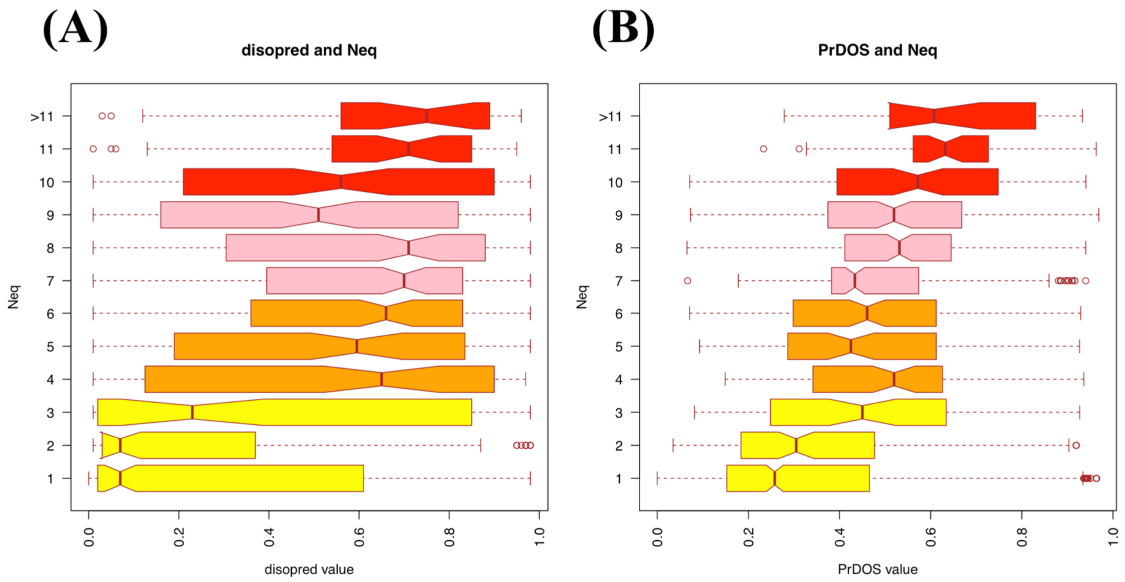

Figure S3. Analyses of prediction disorder results on PED[3] dataset per class of N_{eq}. (A) DisoPred3 values (x-axis) and N_{eq} classes (y-axis). (B) PrDOS values (x-axis) and N_{eq} classes (y-axis).

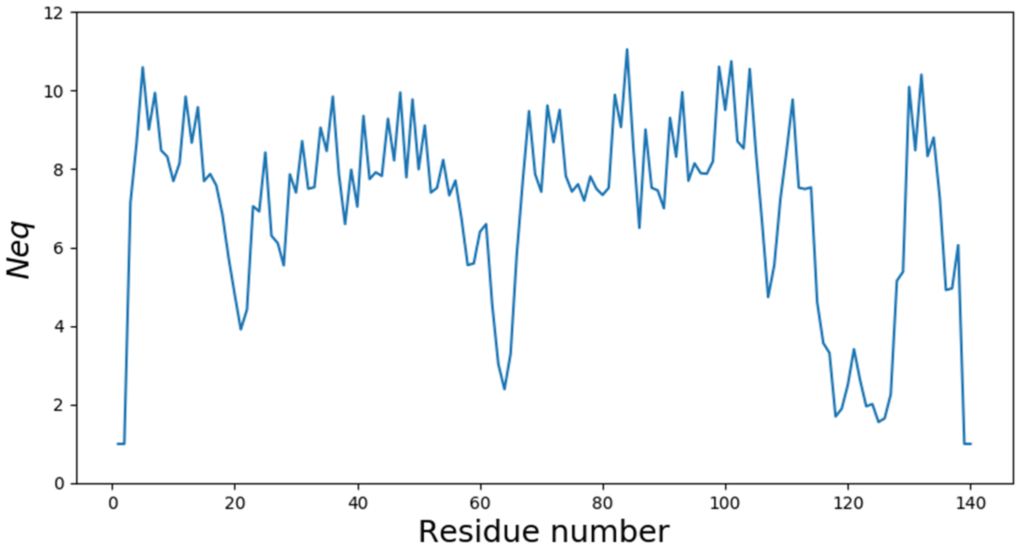

Figure S4. N_{eq} values for the alpha-synuclein, a solution-state ensemble from PRE-NMR ensemble-restrained MD simulations (PED[3] 9AAC entry). Computation done with PBxplore software.

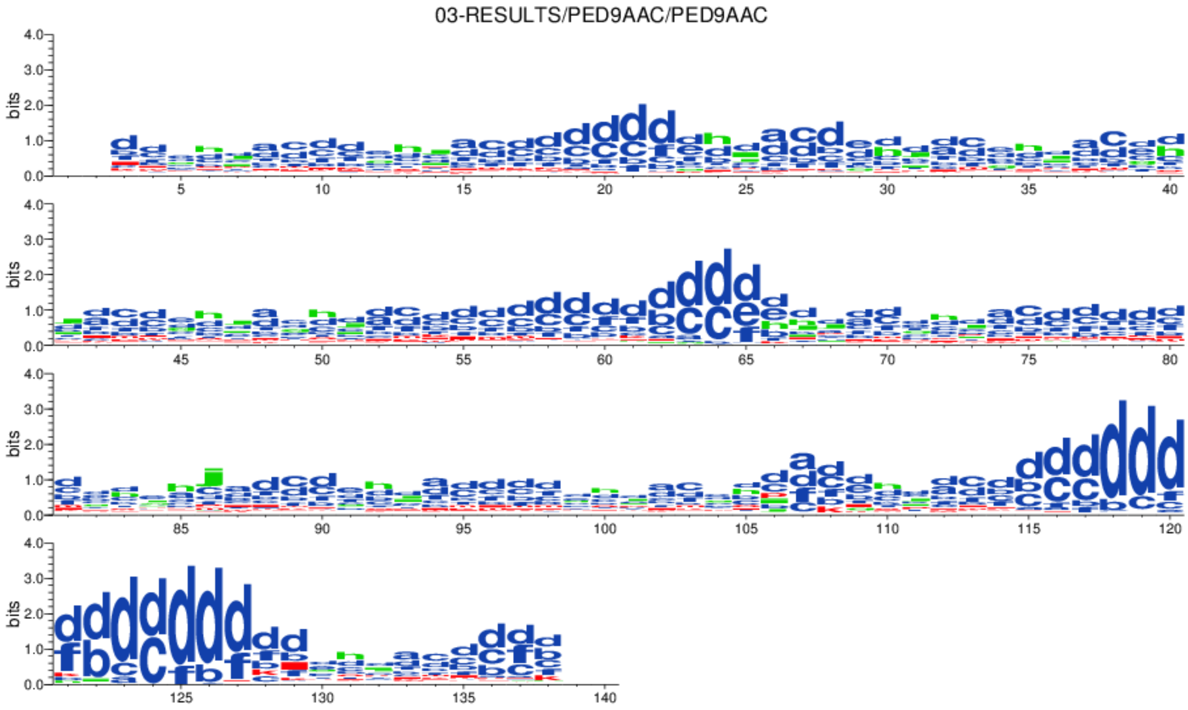

Figure S5. PB distribution for the alpha-synuclein, a solution-state ensemble from PRE-NMR ensemble- restrained MD simulations (PED[3] 9AAC entry). Computation done with PBxplore software and represented with WebLogo.

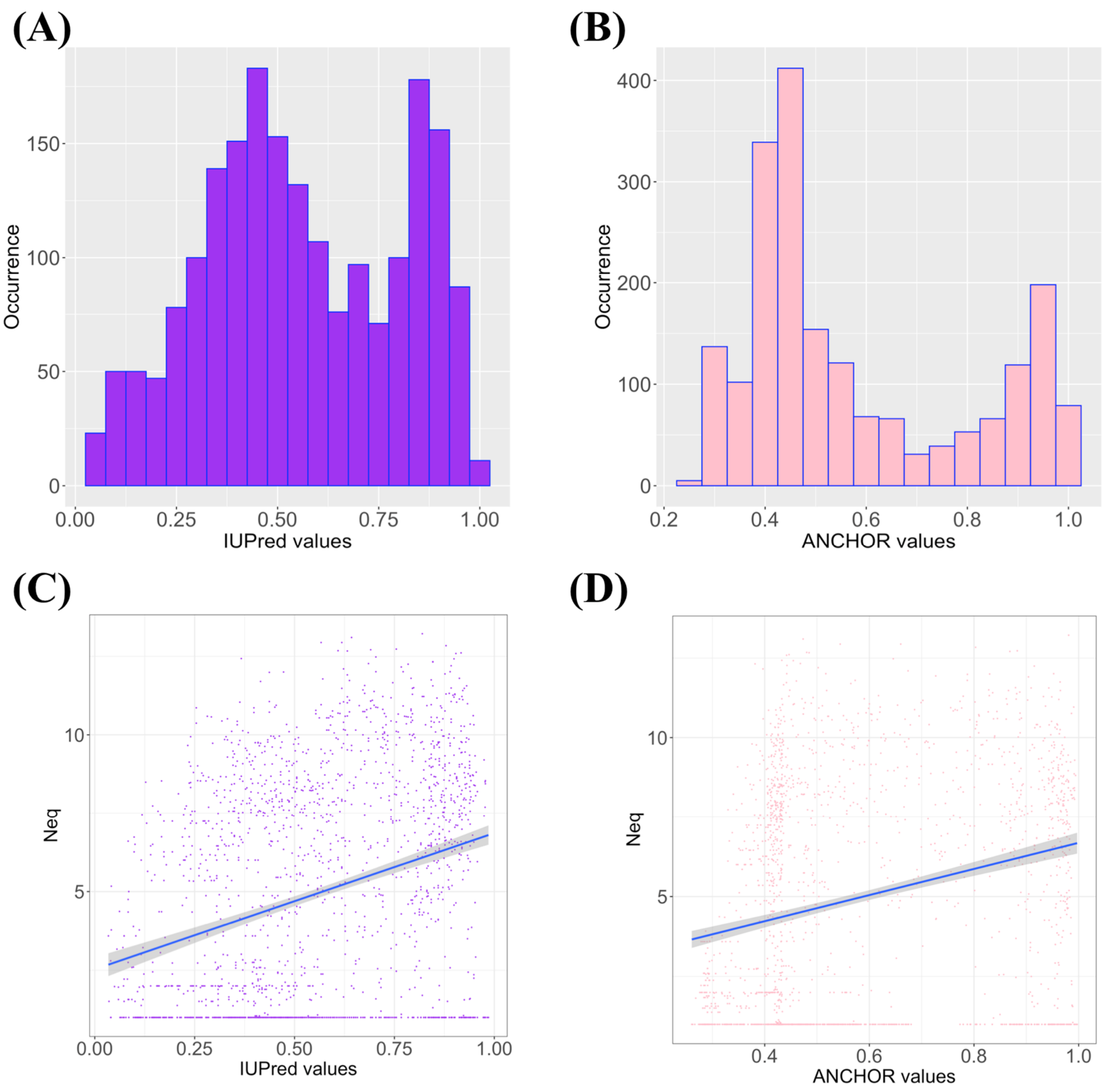

Figure S6. Distribution of N_{eq} and prediction disorder values & N_{eq} versus prediction disorder results. Distribution of (A) IUPred2A values (in purple), and (B) ANCHOR2 values (in pink), (C) N_{eq} values (x-axis) against IUPred2A values (y-axis) (correlation equals to 0.29). (C) N_{eq} values (x-axis) against ANCHOR2 values (y-axis) (correlation equals to 0.25).

	N_{eq}	DisoPred	PrDOS	IUPred2A	ANCHOR2
N_{eq}	--				
DisoPred	**0.37**	--			
PrDOS	**0.34**	***0.75***	--		
IUPred2A	**0.29**	0.56	0.65	--	
ANCHOR2	**0.25**	0.49	0.44	***0.79***	--

Table S1. Correlation between N_{eq}, Disopred3, PrDOS, IUPRed2A and ANCHOR2 values on PED[3] dataset.

Neq	1.0	>2.0	>3.0	>4.0	>5.0	>6.0	>7.0	>8.0	>9.0	>10.0	>11.0	>12.0
Prediction	**61.39**	**42.43**	**38.71**	**38.36**	**38.51**	**37.31**	**36.65**	**34.39**	**34.99**	**35.14**	**36.40**	**37.96**
Order	100.00	55.61	46.68	43.57	39.97	34.89	27.85	17.69	10.65	4.50	1.23	0.49
Disorder	0.00	21.48	26.04	30.08	36.20	41.15	50.65	60.94	73.70	83.85	92.32	97.53

Table S2. Evaluation of prediction rate according to N_{eq} values for Disopred3 approach.
